# Study of temporal variability of salivary cortisol and cortisone by LC-MS/MS using a new atmospheric pressure ionization source

**DOI:** 10.1038/s41598-019-55571-3

**Published:** 2019-12-17

**Authors:** Jelena Bakusic, Siemon De Nys, Matteo Creta, Lode Godderis, Radu Corneliu Duca

**Affiliations:** 1 Environment and Health, Department of Public Health and Primary Care, KU Leuven (University of Leuven), Kapucijnenvoer 35, 3000 Leuven, Belgium; 2 KU Leuven (University of Leuven), Department of Oral Health Sciences, BIOMAT & University Hospitals Leuven (UZ Leuven), Dentistry, Kapucijnenvoer 7, 3000 Leuven, Belgium; 3 IDEWE, External Service for Prevention and Protection at Work, Heverlee, Belgium; 4 Unit Environmental Hygiene and Human Biological Monitoring, Department of Health Protection, National Health Laboratory (LNS), 1, Rue Louis Rech, L-3555 Dudelange, Luxembourg

**Keywords:** Diagnostic markers, Liquid chromatography

## Abstract

There is a growing interest concerning the relevance of salivary cortisone levels in stress-related research. However, studies investigating morning patterns and day-to-day variability of cortisone versus cortisol levels are lacking. Cortisol and cortisone analysis by liquid chromatography-tandem mass spectroscopy (LC-MS/MS) has been widely used for routine laboratory measurements in the last years. The aim of this study was to develop an ultra-performance LC-MS/MS method for the simultaneous quantification of salivary cortisol and cortisone levels for assessing the temporal variability of these hormones. Saliva samples were collected from 18 healthy volunteers at 0, 15, and 30 min after awakening on each day for 1 week and analysed with the newly developed method. We used a novel atmospheric pressure ionization source, which resulted in high sensitivity and specificity for both cortisol and cortisone as well as higher peak values and signal-to-noise ratio as compared with the electrospray ionization source. Cortisone showed similar morning patterns as cortisol: a 25% and 49% increase in levels at 15 and 30 min after awakening, respectively. Most cortisone indices showed somewhat lower day-to-day variability and were less affected by state-related covariates. We recommend further exploration of the potential of salivary cortisone as a biomarker in stress-related research.

## Introduction

Cortisol is a steroid hormone produced by the hypothalamic-pituitary-adrenal (HPA) axis, one of the main components in individuals’ response to stressors. A physiological response of the HPA axis to a stressful event is characterised by a quick increase in cortisol level, followed by a decrease once the stressor is gone^1^. Basal and stress-induced cortisol levels are controlled by the self-regulatory system of the HPA axis, and disturbances in this regulation have been linked to pathophysiological processes in the development of various stress-related body and mental disorders^2^.

The secretion of cortisol follows a circadian rhythm with a sharp increase within the first 30 to 45 min after awakening (cortisol awakening response [CAR]) and a gradual decrease throughout the rest of the day^3^. The CAR has attracted considerable interest since its initial systematic description 20 years ago by Pruessner^3^ and is one of the most commonly used measures of cortisol. Even though the physiological function of CAR has not yet been completely revealed, its potential role in preparing the individual to deal with the upcoming daily demands has repeatedly been emphasized^4^.

In epidemiological studies, CAR is routinely measured by using saliva as a matrix for cortisol assessment^5^. Salivary cortisol level closely approximates the biologically active, unbound plasma fraction and thus is associated with the biological activity of the hormone^6,7^. Another advantage is the ease of sampling, because a typical schedule for CAR involves collecting several samples: a first sample immediately after awakening followed by repeated assessments (at 10- or 15-min intervals) over the next 30 to 60 min^8^. Consequently, salivary CAR assessment has several advantages because of its simplicity and non-invasiveness, which allows for easy self-performed collection of samples at multiple times during the day. Hence, it is the sampling method of choice in epidemiological studies^9^.

However, there are no established norms for CAR, for neither salivary cortisol level immediately after awakening nor peak levels released 30 min after awakening. The reason for this is mainly the relatively high day-to-day variability of CAR^10^ as well as numerous confounding factors, such as age, sex, awakening time, effect of light, weekday versus weekend collection and participant adherence^11^.

Another possible reason for the poor agreement among the reported CAR in healthy individuals is the variety of analytical methods used for quantifying salivary cortisol level^12^. The most commonly used methods in the routine laboratory assessment of salivary cortisol are antibody-based immunoassays, which are relatively sensitive and can be rapidly and easily performed. In addition, cortisol analysis by liquid chromatography coupled with tandem mass spectroscopy (LC-MS/MS) has been widely used for routine laboratory measurement of steroids in the last 15 years because it allows for simultaneous quantification of multiple compounds with high sensitivity and specificity and wide dynamic range^13,14^. From the studies comparing the two methods, most immunoassays overestimated absolute cortisol levels in saliva as compared with data obtained by mass spectrometry as a reference method^12,15^, likely because of the cross-reactivity in immunoassays with cortisol metabolites (cortisone) or exogenous glucocorticoids^16^. Nevertheless, recent studies suggested that novel immunoassays can have comparable specificity to that obtained with LC-MS/MS^17^ and that the two methods are largely comparable in interpreting salivary cortisol dynamics in stress research^15^. Finally, LC–MS/MS methods showed better comparability than immunoassays among laboratories^15^. Consequently, there is a number of developed methods for assessing salivary cortisol level with LC-MS/MS in the literature, mostly using the electrospray ionisation (ESI) source^13,18^.

The cortisol: cortisone ratio in saliva is opposite to that in plasma. Plasma cortisol: cortisone ratios are approximately 8:1, and salivary cortisone level is 2 to 6 times higher than cortisol level. This difference is caused by the conversion of the unbound fraction of cortisol to cortisone by the enzyme 11ß-hydroxysteroid dehydrogenase 2 (11ß-HSD2) when passing the salivary glands^19^. Therefore, the relevance of salivary cortisone in stress-related research has received much interest, especially since salivary cortisone has been found strongly associated with serum cortisol levels^20,21^. Furthermore, a recent study showed salivary cortisone level with more potential as a stress biomarker than cortisol level because of a higher discriminatory power and significant association with subjective and autonomic stress measures in healthy individuals^22^. Nevertheless, more research is necessary to evaluate the potential of salivary cortisone as a biomarker of stress, especially with the lack of exploration of morning patterns of salivary cortisone (repeated measurements in the first hour after awakening). In addition, we lack assessment of day-to-day variability of salivary cortisone level in healthy individuals and its comparison with cortisol levels.

In the present study, we aimed to develop a sensitive and specific ultra-performance LC (UPLC)-MS/MS method for the simultaneous identification and quantification of salivary cortisol and cortisone to assess the temporal variability of these hormones in healthy individuals over 1 week.

## Materials and Methods

### Chemicals and materials

Cortisol and cortisone, d4-cortisol and d8-cortisone internal standards (ISs) and LC-MS-grade methanol and formic acid were purchased from Sigma-Aldrich (Diegem, Belgium). LC-MS grade water was obtained from Biosolve (Valkenswaard, The Netherlands). Cotton Salivettes® (Cortisol, code blue) for saliva collection were provided by Sarstedt (Nümbrecht, Germany) and 1.5 mL polypropylene tubes were from Eppendorf (Hamburg, Germany). Oasis PRiME HLB extraction cartridges (3 mL; 60 mg) were purchased from Waters (Zellik, Belgium).

Cortisol and cortisone standards were dissolved in methanol to obtain primary stock solutions of 5 mg/mL and 10 mg/mL, respectively, and were stored at −80 °C. Secondary stock solutions were obtained by dissolving the primary stock solutions in methanol to obtain concentrations of 50 and 100 µg/mL, respectively, and were stored at −20 °C for up to 3 months. Calibration dilutions containing cortisol in a concentration of 20 ng/mL and cortisone in a concentration of 10 ng/mL were obtained by diluting the secondary stock solutions in water and were prepared fresh during sample preparation.

D4-cortisol and d8-cortisone ISs were dissolved in methanol to obtain primary stock solutions of 5 and 1 mg/mL, respectively. These solutions were stored at −80 °C. Secondary IS stock solutions were obtained by dissolving the primary stock solutions in methanol to obtain concentrations of 50 and 10 µg/mL, respectively, which were stored at −20 °C for up to 3 months. IS solutions containing both ISs at 1 µg/mL were obtained by diluting the secondary stock solutions in water and were prepared fresh before sample and calibration preparation.

### Studied population and sample collection

We recruited 18 healthy volunteers (10 females) 23 to 39 years old among the KU Leuven staff after they gave informed consent to be in the study according to the Helsinki declaration. The study was approved by the University Hospital Leuven Medical Ethics Committee (S59567). All participants were asked to collect three saliva samples in the morning: immediately after awakening and 15 and 30 min thereafter and to repeat the procedure every day for 1 regular working week (including the weekend). Participants were asked to avoid drinking, eating and brushing their teeth before the collection of all three saliva specimens. Smokers were asked to refrain from smoking before collecting all three samples. All participants were asked to report the exact time of sample collection and potential covariates that could affect cortisol and cortisone levels: duration of sleep, time of awakening, alcohol consumption, smoking, medication use and whether sampling was performed on a working or non-working day^8^. The median reported duration of sleep was 8 hr (min-max: 5–12 hr) and the median time of awakening was 7.50 AM (min-max: 3.30 AM-1 PM). Three participants were smokers and all reported smoking 5 cigarettes per day on average. Alcohol intake for each day was calculated based on the information reported in diaries on the type and volume of beverage and was expressed in units^23^. Most participants did not consume any alcohol in the period of sample collection. Six participants reported taking anti-inflammatory medication occasionally (ibuprofen, paracetamol or aspirin). Medication use was scored as a dichotomous variable (yes/no) for each day separately. Two participants reported taking oral contraceptives.

All specimens were collected between March and May 2017 to avoid a potential seasonal effect on cortisol and cortisone levels^8^. All samples were stored at 4 °C until delivery to the laboratory, where samples were immediately centrifuged at 2000 x g for 5 min to obtain clear saliva with low viscosity. Samples were then transferred to 1.5-mL tubes and stored at −80 °C.

### Sample preparation

To purify cortisol and cortisone and remove as much as possible impurities from the saliva that could interfere with MS analysis, solid-phase extraction was performed by using a slightly modified method previously described by Antonelli^24^. Each saliva sample or calibrator (200 µL) was spiked with IS calibration solution (20 µL) and diluted with 800 µL water. The samples and calibrators were then applied to Oasis PRiME HLB solid-phase extraction cartridges (3 mL; 60 mg). After sample loading (1 mL), the washing step was performed with 500 µL water:methanol (95:5, v/v), followed by two extraction steps with 500 µL methanol. The eluates were evaporated to dryness under a gentle nitrogen flow and reconstituted in 1 mL water:methanol (50:50, v/v) containing 0.1% formic acid. Finally, 10 µL of samples was injected into the UPLC-MS/MS system.

### Apparatus and operating conditions

A Xevo TQ-XS Tandem Quadrupole Mass Spectrometer (Waters, Zellik, Belgium) equipped with an ESI or UniSpray ionization (USI) source was used for all sample analyses.

The ESI source is one of the most-used atmospheric pressure ionization sources; the electrospray is produced by applying a strong electric field to the analyte solution passing through a capillary tube. The ESI source temperature was set at 150 °C and the desolvation temperature 600 °C. Argon was used as the collision gas (0.5 mL/min) and nitrogen as the desolvation gas (1000 L/hr). Nitrogen was also used as nebulization gas (7 bar).

Unlike ESI, the USI does not have a voltage applied on the capillary tip through which the analyte solution passes. The USI is a novel atmospheric pressure ionization source whereby the eluent spray impacts on a cylindrical, stainless steel target rod held at high voltage. The impact point is optimized to be offset from the centre of the rod, which allows the flow of the eluent spray to bend around the profile of the rod because of the Coandă effect toward the MS inlet. USI source temperature was set at 150 °C and desolvation temperature 500 °C. Argon was used as the collision gas and nitrogen for desolvation and nebulisation (the same parameters as when ESI was applied). Multiple reaction monitoring (MRM) parameters for the analysis of levels of cortisol, cortisone and the ISs are summarized in Table 1. For each compound, the MRM transition with the highest measured response was set as the “quantifier transition”. Other transitions were used as “qualifier transitions” for confirmation. Analysis was performed in positive ionization mode.

**Table 1:** MS/MS parameters for the analysis of target compounds cortisol and cortisone and the internal standards d4-cortisol and d-8 cortisone.

Compound	R_t_	Ionization	Quantifier			Qualifier		
	(min)	mode	Transition	Cone/Impactor* (V)	Collision (V)	Transition	Cone/impactor*(V)	Collision (V)
Cortisol	1.84	ESI+	363.3 → 120.6	32	22	363.3 → 90.5	32	54
*d4-Cortisol*	1.85	ESI+	367.3 → 120.7	26	20			
Cortisone	1.75	ESI+	361.2 → 162.8	26	24	361.2 → 104.6	26	40
*d8-Cortisone*	1.73	ESI+	369.3 → 168.8	34	24			
Cortisol	1.84	USI+	363.2 → 120.7	34	26	363.2 → 327.2	34	16
*d4-Cortisol*	1.84	USI+	367.2 → 120.7	38	24			
Cortisone	1.74	USI+	361.2 → 162.8	38	24	361.2 → 104.6	38	46
*d8-Cortisone*	1.73	USI+	369.3 → 168.9	50	28			

Rt: retention time; V: voltage; *for USI source the ionization involves a stainless steel rod. ESI, electrospray ionization; UNI, UniSpray ionization.

The mixture of samples or calibrators (1 mL) was injected in an Acquity UPLC BEH C18 column (50 mm × 2.1 mm, 1.7 µm, Waters), kept at 40 °C. An optimal chromatographic separation was achieved by using a mixture of H_2_O containing 0.1% formic acid (solvent A) and MeOH containing 0.1% formic acid (solvent B) as a mobile phase, with the following gradient: 0 to 2 min, 35% B; 2 to 2.7 min, 95% B; 2.7 to 3.2 min, 35% B. The flow rate was set at 0.4 mL/min.

### Method performance and validation

The developed method was validated for linearity of calibration curves and the associated correlation coefficient R², limit of detection (LOD), limit of quantification (LOQ), and intra- and inter-assay accuracy and precision, as we previously described^25^.

#### Linearity and limits of quantification

To test the matrix effect of saliva, two calibrations were prepared, the first in the synthetic matrix and the second in the biological matrix (late-night saliva). For the first calibration, serial dilutions were prepared in a synthetic matrix containing water:methanol (50:50, v/v) and 0.1% formic acid, in 10 concentrations ranging from 0.01 to 10 ng/mL for cortisol and 0.005 to 5 ng/mL for cortisone. All calibrators were spiked with the IS solution, with the final concentration of 20 ng/mL. For the second calibration, a pool of Salivette®-specimens, collected late at night to obtain the lowest possible levels of cortisol and cortisone, was used to prepare spiked calibration dilutions with a wide range of concentrations. Cortisol levels in late-night saliva were undetectable whereas the analyte peak area for cortisone detected in the unspiked saliva was subtracted from the peak area of rest of the calibration points. The specimens were analysed by preparing pentaplicates spiked with known amounts of cortisol and cortisone, in 10 concentrations from 0.01 to 10 ng/mL for cortisol and 0.005 to 5 ng/mL for cortisone, which is a wider range then the expected morning concentrations for the two compounds in real saliva samples. All calibrators were spiked with the IS solution to obtain the concentration of 20 ng/mL and were freshly prepared and analysed the same day. For both calibrations, the calibration curves were fitted by linear regression analysis on the peak area ratios of the target compounds to the IS, against the different nominal concentrations of the target compounds. The fitting of the calibration curves was evaluated by using the error on the calculated concentrations expressed as percentage of target concentrations (% of target) and the correlation coefficient R² of the calibration curves. The sample concentrations were calculated from the equation y = mx + b, as determined by weighted (1/x^2^) linear regression of the standard line. Moreover, slopes of the standard lines obtained for the calibration in standard dilutions and that in saliva were compared to evaluate the matrix effect of saliva, as suggested by Matuszewski^26^. The LOD was defined as the lowest concentration producing a peak with a signal-to-noise ratio (S/N) ≥ 3. The LOQ was defined as the lowest concentration producing a peak with an S/N ≥ 10 measurable with 85% to 115% accuracy and imprecision ≤15%.

#### Intra-assay and inter-assay accuracy and precision

To ensure correct quantification, intra-assay accuracy and precision were calculated. For calibration in synthetic matrix, these values were evaluated by injecting five times the calibrators spiked at low, medium and high concentration (1.4, 6 and 10 ng/mL for cortisol and 0.7, 3 and 5 ng/mL for cortisone), whereas for calibration in biological matrix (late-night saliva), these values were obtained by analysing prepared pentaplicates of the three calibration points spiked at the same low, medium and high levels. The intra-assay accuracy for each calibrator was calculated as the error to the nominal concentrations (% of target). The intra-assay precision was calculated as the relative standard deviation (RSD%). Inter-assay accuracy and precision were assessed by analysing 15 replicates of the spiked calibrators or saliva pool specimens on different days. The precision determined at each concentration should not exceed 15% and the accuracy should be 85% to 115%.

#### Selectivity

All target compounds were analysed by using compound-specific MRM, for a reduction of interference from other compounds in the sample. Moreover, besides a quantification transition (“quantifier”), at least one additional transition (“qualifier”) was used for each of the target compounds. Additionally, correct identification of target compounds was ensured by checking the target-specific retention times (Table 1).

### Statistical analysis

To estimate CAR, we calculated the standardized total area under the curve (AUC). We used two equations for AUC with respect to increase (AUCi) and AUC with respect to ground (AUCg), as reported by Pruessner^27^.

A linear mixed-model was fitted for cortisol and cortisone levels, with one random intercept to capture the correlation among measurements within the same participant. To investigate which confounding factors affected cortisol and cortisone levels, we developed separate unadjusted linear mixed-effects models for each variable of interest. The following model was fitted:

$${Y}_{ij}={\beta }_{0}+{\mu }_{0}+{\beta }_{1}{X}_{1}+{\varepsilon }_{ij}$$

where *Y*_*ij*_ is the measured cortisol or cortisone level of participant *j* on day *i*; β_0_ is the overall intercept, μ_0_ is the random intercept, and X_1_ is a confounding factor. The following *a priori*-chosen confounding factors from the literature^8^, were tested in the univariate models: age, sex, working day, duration of sleep, time of awakening, smoking, alcohol consumption, use of medication and use of oral contraceptives.

To investigate the temporal variability of cortisol and cortisone measurements over 1 week, we fitted a model with the intercept and the random effect of the individual only. We used this model to calculate the intraclass correlation coefficient (ICC), an estimator of the proportion of between-unit variance to total variance^28^. In this context, ICC gives an indication of the day-to-day variability of cortisol and cortisone measurements within the same person and can be interpreted as the expected correlation between two randomly chosen samples of the same individual on 2 different days^29^. Therefore, the ICC was as follows:

$${\rm{ICC}}={{{\rm{\sigma }}}_{{\rm{p}}}}^{2}/{{{\rm{\sigma }}}_{{\rm{p}}}}^{2}+{{{\rm{\sigma }}}_{{\rm{e}}}}^{2}$$

where σ_p_^2^ represents variance systemically accounting for the level of “persons” and σ_e_^2^ quantifies the residual variance, which is mainly reflected by within-participant or day-to-day variability.

ICC values range from 0 to 1, with 0 indicating lack of any correlation between two measurements and 1 implicating that the two measurements are identical^29^. ICC values 0 to 0.5 are considered to indicate low stability, 0.5 to 0.75 moderate stability, and 0.75 to 1 high stability of observed measurements.

R 3.3.0 (R Foundation for Statistical Computing, Vienna, Austria) was used for statistical analyses and the level of significance was set at α < 0.05.

## Results

### Method development and validation

#### Optimisation of the UPLC-MS/MS operating conditions

The UPLC-MS/MS method was developed by optimising the following segments: 1) MS conditions, 2) chromatographic conditions, and 3) sample preparation.

**Figure 1:**
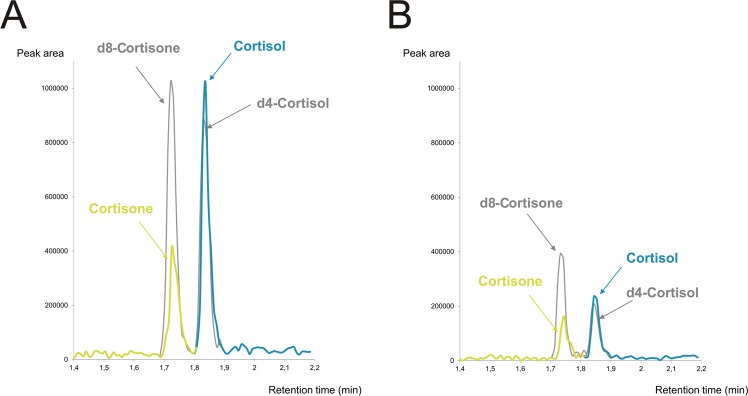
Comparison of chromatograms for cortisol, cortisone and internal standards, obtained with UniSpray ionization (**A**) and electrospray ionization (**B**). Concentration equals 1 ng/mL for all compounds.

Optimisation of the MS parameters was achieved by direct infusion of the standard solutions of cortisol and cortisone into the ESI and USI sources at flow rate 20 μL/min. The MS parameters optimised included source temperature, desolvation temperature, and gas flows. For each target compound, the most sensitive MRM transition was selected for each ionisation source as a quantifier, and one additional transition was selected as a confirmative determinant (qualifier). A detailed description of the optimal MS/MS conditions is in Table 1. Moreover, we compared the Limit of detection (LOD) and Limit of quantification (LOQ) values and the chromatograms obtained using the new USI source with the commonly used ESI source. Use of ESI source resulted in higher LOD (100 pg/mL for cortisol and 500 pg/mL for cortisone) and LOQ (500 pg/mL for cortisol and 1 ng/mL for cortisone) compared to those obtained using the new USI source (LOD = 5 pg/mL for both compounds, LOQ = 10 pg/mL for cortisol and LOQ = 50 pg/mL for cortisone). To test differences in the pick values and S/N ratio, we compared chromatograms obtained using ESI and USI source for the concentration of 1 ng/mL of all compounds (which equals to the LOQ obtained with the ESI source). Use of the USI source resulted in higher peak values and higher S/N ratio for both cortisol (peak height = 377664, S/N = 158.4) and cortisone (peak height = 473117, S/N = 299.4) as compared with ESI (cortisol: peak height = 94614, S/N = 51.4, cortisone: peak height = 56463, S/N = 32.7). Examples of chromatograms obtained with both sources for the both compounds and ISs (C = 1 ng/mL for all compounds) is in Fig. 1.

Chromatographic conditions were adjusted to achieve optimal resolution, peak shape, and enhanced response. Moreover, additional efforts were made to ensure efficient LC separation because the retention time of a compound, together with the MS transition, is the main parameter that guarantees absolute specificity for the identified compound. Modifications of the solvent flow rate (from 200 to 500 μL/min) and the separation gradient had a much smaller effect on the chromatographic separation as compared with the buffer type (data not shown). Therefore, we focused more on testing the impact of different mobile phases on the chromatogram. For instance, use of a mixture containing acetonitrile containing 0.1% formic acid and H_2_O containing 0.1% formic acid in different gradient concentrations resulted in poor chromatographic parameters. Unlike acetonitrile, the use of MeOH containing 0.1% formic acid combined with H_2_O containing 0.1% formic acid resulted in optimal chromatographic parameters and satisfactory LOQ. Moreover, we tested three types of columns to obtain optimal LC separation of the target compounds: an Acquity UPLC BEH C18 column (50 mm × 2.1 mm, 1.7 µm, Waters), a hydrophilic interaction LC (HILIC) column (Phenomenex® Kinetex 2.6 μm Hilic, 50 × 4.6 mm), and an Acquity UPLC BEH Amide column (50 × 2.1 mm, 1.7 µm, Waters). Test samples analysed with the Acquity UPLC BEH C18 column displayed the highest peak values and the lowest background noise and it was therefore used for further analysis (data not shown).

Finally, during the method development, we tested three different extraction methods: solid-phase extraction with the Oasis PRiME HLB extraction cartridges (3 mL; 60 mg) (Zellik, Belgium), solid liquid extraction with the NOVUM extraction cartridge (Phenomenex, Belgium), and liquid–liquid extraction with dichloromethane. For each method, we prepared three test samples spiked with 5 µg/mL cortisol before extraction and three samples spiked with the same concentration after the extraction, right before injection. We tested recovery of cortisol by dividing the average peak area of the samples spiked after extraction by the average response of the pre-spiked samples and multiplied the result by 100 to obtain the recovery percentage^26^. The highest recovery was obtained by applying the solid-phase extraction method with the Oasis PRiME HLB cartridge (98 ± 13%) as compared with the NOVUM solid-liquid extraction cartridge (9 ± 2%), and liquid–liquid extraction (66 ± 25%). Therefore, the solid-phase extraction method using the Oasis PRiME HLB cartridge was used.

#### Linearity and limits of quantification

The correlation coefficients from the regression equations were 0.999 for cortisol (y = 7 × 10^−5^x − 0.0003) and 0.998 for cortisone (y = 7 × 10^−5^x + 0.0067) for calibration in the synthetic matrix and 0.999 for cortisol (y = 7 × 10^−5^x + 0.0024) and 0.996 for cortisone (y = 7 × 10^−5^x + 0.0516) for calibration in the biological matrix (late-night saliva). Comparison of slopes from the regression equations revealed minimal matrix effect of saliva on both cortisol and cortisone. However, the biological (salivary) matrix resulted in an increase in the LOD from 5 to 20 pg/mL, for both compounds. Similarly, LOQ for cortisol increased from 10 pg/mL in the synthetic matrix to 100 pg/mL in the salivary matrix, but the LOQ for cortisone remained unchanged (50 pg/mL). Both instrumental (synthetic matrix) and procedural (biological matrix) LOD and LOQ values are in Table 2. Appropriate values for both functional accuracy and precision were obtained for all standard dilutions and saliva pool specimens spiked with a wide range of concentrations for both compounds, as presented in Table 2. These results indicate good correlation between the measured response (peak area) and the nominal concentrations of cortisol and cortisone.

**Table 2:** Instrumental (synthetic matrix) and procedural (salivary matrix) validation parameters.

Compound	Instrumental parameters		Procedural parameters						
	LOD ng/mL (nmol/L)	LOQ ng/mL (nmol/L)	LOD ng/mL (nmol/L)	LOQ ng/mL (nmol/L)	Tested Concentrations ng/mL (nmol/L)	Intra-assay		Inter-assay	
						Accuracy (% of target) (n = 5)	Precision (RSD%) (n = 5)	Accuracy (% of target) (n = 15)	Precision (RSD%) (n = 15)
Cortisol	0.005 (0.014)	0.01 (0.028)	0.02 (0.055)	0.1 (0.276)	1.4 (3.9)	95	3	94	4
					6 (16.6)	96	3	94	3
					10 (27.6)	97	2	95	3
Cortisone	0.005 (0.014)	0.05 (0.138)	0.02 (0.055)	0.05 (0.138)	0.7 (1.9)	98	4	105	7
					3 (8.3)	99	3	101	4
					5 (13.8)	103	3	105	4

LOD: limit of detection; LOQ: limit of quantification; RSD: relative standard deviation.

#### Intra- and inter-assay accuracy and precision

The intra-assay accuracy was 95% to 97% for cortisol and 98% to 103% for cortisone. The precision range for cortisol was 2% to 3% RSD, whereas the precision range for the cortisone concentrations was 3% to 4% RSD. Inter-assay accuracy for both compounds ranged from 94% to 105%, and intra-assay precision did not exceed 7% (Table 2).

### Cortisol and cortisone morning patterns

**Figure 2:**
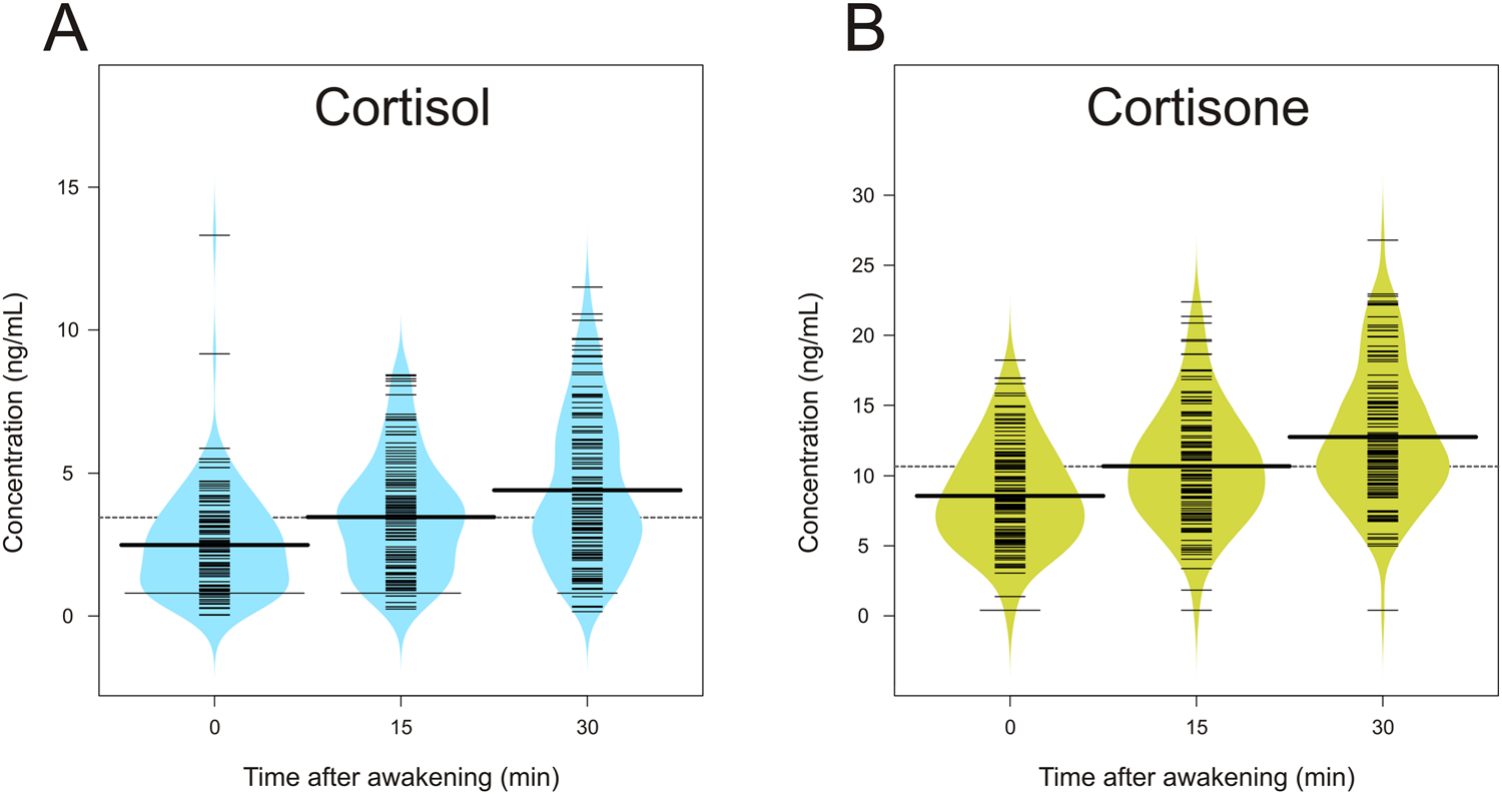
Average cortisol (**A**) and cortisone (**B**) levels in the overall sample at 0, 15 and 30 min after awakening.

**Figure 3:**
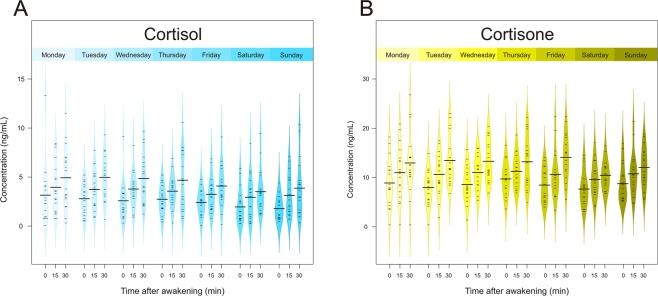
Average cortisol (**A**) and cortisone (**B**) levels at 0, 15 and 30 min after awakening on each day over 1 sampling week.

The average concentration of morning cortisol showed the expected increase from 0 to 30 min after awakening. The relative increase from the baseline concentration on awakening (2.49 ± 1.83 ng/mL, equivalent to 6.87 ± 5.05 nmol/L) was 39% after 15 min (3.46 ± 2 ng/mL, equivalent to 9.54 ± 5.52 nmol/L) and reached 77% at the 30-min peak (4.4 ± 2.6 ng/mL, equivalent to 12.14 ± 7.17 nmol/L). However average concentrations of cortisone were somewhat higher but showed a slightly lower relative increase in the first 30 min after awakening. The average baseline concentration of cortisone right after awakening was 8.57 ± 3.66 ng/mL (equivalent to 23.74 ± 10.14 nmol/L), with a 25% increase after 15 min (10.68 ± 4.12 ng/mL, equivalent to 29.58 ± 11.41 nmol/L) and 49% higher than the baseline value at 30 min after awakening (12.77 ± 4.74 ng/mL, equivalent to 35.37 ± 13.13 nmol/L). The mean values of the aggregate measures of cortisol were 6.9 ± 3.44 ng/mL (AUCg) and 1.94 ± 3.39 ng/mL (AUCi), whereas the same values for cortisone were 21.31 ± 7.22 ng/mL (AUCg) and 4.2 ± 5.93 ng/mL (AUCi). Figure 2 gives an overview of the mean values of single-sample measures (awakening, 15 and 30 min) for cortisol and cortisone in the total sample, and Fig. 3 depicts the mean concentrations of cortisol and cortisone measurements for each day of the week. Also, Supplementary Fig. 1 gives an overview of individual cortisol and cortisone levels for each participant.

### Effect of covariates on cortisol and cortisone levels

We tested the effect of each covariate on all single and aggregate measures of cortisol and cortisone by univariate analysis. An overview of the effects of covariates on cortisol and cortisone single and aggregate measures is in Table 3. First, time of awakening had a significant negative effect on most of the cortisol indices. More specifically, for every hour of earlier awakening time, cortisol level increased 0.24 units (of standard deviation) at 0 min (Χ^2^ = −0.24, p = 0.0299), 0.36 units at 15 min (Χ^2^ = −0.36, p = 0.0052) and 0.42 units at 30 min after awakening (Χ^2^ = −0.42, p = 0.0037). In addition, a 0.6-unit increase in cortisol AUCg corresponded to a 1-hr decrease in the awakening time (Χ^2^ = −0.6, p = 0.001). Second, as compared with the levels measured on the weekend, measurements on a working day showed cortisol concentration increased 0.83 units at awakening time (Χ^2^ = −0.83, p = 0.0106), 0.92 units at 30 min after awakening (Χ^2^ = −0.92, p = 0.0227) and 1.35 units for the cortisol AUCg (Χ^2^ = −1.35, p = 0.0195). Finally, medication use had a significant positive impact on cortisol levels at 30 min after awakening (Χ^2^ = 1.65, p = 0.0148) and AUCg (Χ^2^ = 2.55, p = 0.005), whereas smoking decreased cortisol AUCg (Χ^2^ = −2.97, p = 0.0183). Cortisol AUCi was not affected by any of the covariates.

**Table 3:** Overview of the effects of covariates on cortisol and cortisone levels.

	Time of measurement after awakening			AUCg	AUCi
	0 min	15 min	30 min		
**Cortisol**					
Working day	−0.83 (0.32)*	**−**0.49 (0.35)	−0.92 (0.4)*	−1.35 (0.57)*	0.27 (0.56)
Duration of sleep	**−**0.15 (0.14)	**−**0.2 (0.15)	**−**0.23 (0.18)	**−**0.43 (0.26)	**−**0.02 (0.26)
Time of awakening	−0.24 (0.12)*	−0.36 (0.12)**	−0.42 (0.12)**	−0.6 (0.18)**	**−**0.12 (0.18)
Medication use	0.86 (0.46)	0.88 (0.52)	1.65 (0.66)*	2.55 (0.89)**	0.74 (0.9)
Smoking	**−**1.11 (0.59)	−1.47 (0.6)*	**−**1.86 (1.07)	−2.97 (1.13)*	**−**0.72 (1.38)
Sex	0.46 (0.45)	0.24 (0.52)	0.02 (0.88)	0.48 (1.01)	**−**0.42 (1.04)
Age	**−**0.04 (0.05)	0.006 (0.06)	0.03 (0.1)	0.003 (0.12)	0.08 (0.12)
Use of contraceptive pills	**−**0.58 (0.72)	0.28 (0.83)	0.74 (1.38)	0.35 (1.61)	1.53 (1.61)
**Cortisone**					
Working day	**−**0.37 (0.6)	**−**0.52 (0.68)	−1.66 (0.73)*	**−**1.46 (1.13)	**−**0.74 (1.04)
Duration of sleep	**−**0.39 (0.26)	**−**0.24 (0.31)	**−**0.34 (0.33)	**−**0.59 (0.53)	0.05 (0.46)
Time of awakening	**−**0.036 (0.24)	**−**0.3 (0.24)	−0.6 (0.24)*	**−**0.6 (0.42)	−0.72 (0.36)*
Medication use	0.72 (0.99)	2.37 (1.08)*	3.08 (1.21)*	5.36 (1.85)**	2.97 (1.58)
Smoking	−3.09 (1.31)*	−4.64 (1.2)**	−5.68 (1.53)**	−9.1 (2.21)**	**−**2.88 (2.03)
Sex	**−**0.72 (1.12)	**−**1.57 (1.19)	**−**1.92 (1.49)	**−**2.99 (2.26)	**−**1.51 (1.57)
Age	**−**0.04 (0.13)	0.11 (0.14)	0.14 (0.17)	0.17 (0.27)	0.24 (0.17)
Use of contraceptive pills	2.78 (1.66)	3.59 (1.76)	3.11 (2.35)	6.48 (3.39)	0.96 (2.54)

Data are expressed as X^2^ (SE). AUCi, area under the curve with respect to increase; AUCg, area under the curve with respect to ground.

For cortisone measurements, time of awakening negatively affected only the level at 30 min after awakening (Χ^2^ = −0.6, p = 0.017) and AUCi (Χ^2^ = −0.72, p = 0.0465), whereas working day contributed significantly to increased level of cortisone only at 30 min after awakening (Χ^2^ = −1.66, p = 0.0247). Smoking had a significant negative impact on several cortisone measurements: 0 min (Χ^2^ = −3.09, p = 0.031), 15 min (Χ^2^ = −4.64, p = 0.0014) and 30 min after awakening (Χ^2^ = −5.68, p = 0.0019) as well as AUCg (Χ^2^ = −9.1, p = 8e04). Finally, medication use contributed to increased level of cortisone at 15 min (Χ^2^ = 2.37, p = 0.0301) and 30 min after awakening (Χ^2^ = 3.08, p = 0.0125) and AUCg (Χ^2^ = 5.36, p = 0.0045).

Moreover, working day and time of awakening had an interaction effect, which reached statistical significance for most of the cortisol and cortisone measures: cortisone at awakening time (Χ^2^ = −1.8, p = 0.0013), cortisol and cortisone at 15 min (Χ^2^ = −1.2, p = 0.0013 and Χ^2^ = −2.4, p = 0.0003) and 30 min after awakening (Χ^2^ = −1.2, p = 0,0104 and Χ^2^ = −1.8, p = 0.003 respectively) as well as AUCg (cortisol: Χ^2^ = −1.8, p = 0.0027 and cortisone: Χ^2^ = −4.2, p = 0.0001). In other words, working day had a stronger negative impact on cortisol and cortisone levels if participants woke up earlier or vice versa, earlier awakening hours had a stronger negative impact on cortisol and cortisone measurements on a working day than a weekend day.

Duration of sleep, sex, age and use of oral contraceptives did not affect any of the outcome variables.

### Stability of cortisol and cortisone measurements

The ICC values derived from our analyses were from 0.15 to 0.37 for cortisol and somewhat higher for cortisone (0.19–0.36). All ICC values indicated high day-to-day variability and low stability of cortisol and cortisol measurements. The least stable measurement of cortisol was the level on awakening (ICC = 0.15) and 15 min after awakening (ICC = 0.17), whereas levels at 30 min after awakening seemed to be somewhat more stable (ICC = 0.37) as did both aggregate measurements (ICC_AUCi_ = 0.29, ICC_AUCg_ = 0.25). However, single measurements of cortisone showed higher stability as compared with cortisol (ICC_0min_ = 0.3, ICC_15min_ = 0.28, ICC_30min_ = 0.36), whereas the two aggregate measurements for cortisone differed in the ICC values. More specifically, the overall release of cortisone, reflected by AUCg seemed to be more stable over the week (ICC = 0.36) than the morning increase, as reflected by AUCi (ICC = 0.19). Table 4 gives ICC values of cortisol and cortisone measurement indexes.

**Table 4:** Intraclass Correlation Coefficients (ICCs) for single and aggregate measures of cortisol and cortisone.

	Time of measurement after awakening			AUCg	AUCi
	0 min	15 min	30 min		
**Cortisol**					
Interindividual variance	0.51	0.68	2.59	3.05	3.38
Residual variance	2.87	3.34	4.41	8.97	8.38
**ICC (%)**	**14.99**	**16.96**	**36.98**	**25.37**	**28.75**
**Cortisone**					
Interindividual variance	4.06	4.77	8.19	18.74	6.76
Residual variance	9.51	12.41	14.63	34.02	28.76
**ICC (%)**	**29.93**	**27.78**	**35.89**	**35.52**	**19.04**

## Discussion

In the present study, we developed and validated a sensitive and specific UPLC-MS/MS method for simultaneous quantification of salivary cortisol and cortisone. The novelty of the approach lies in the ability to accurately measure these two compounds by using a UPLC-MS/MS system with a novel atmospheric pressure ionization source (i.e., USI) in positive ionisation mode. To our knowledge, this is the first method developed for determining salivary cortisol and cortisone using this source because all previously published methods involved ESI^21,23,30,31^. The first results on the increased performance of the USI source were published recently but were in a context of eicosanaoids analysis^32^ and quantification of pharmaceutical compounds^33^. The results showed a general increase in signal intensity observed with USI as compared with ESI, which resulted in a lower limit of quantification. Similarly, in our study, we observed higher peak values and higher S/N ratio when we used the USI source for both cortisol and cortisone. Therefore, this was our source of choice for the sample analysis.

The specific transitions and retention times detected by our UPLC-MS/MS method enabled a clear distinction between cortisol and its metabolite cortisone, thus enabling a simultaneous assessment of both compounds with high specificity. In a previous publication^34^, low specificity due to cross-reactivity was found as a major limitation of immunoassays. The results showed a lack of linear correlation between cortisol measurements obtained with different immunoassays and LC-MS/MS, and the “nonlinearity” factor was further significantly correlated with the reported cross-reactivity between cortisol and cortisone in analyses with the most frequently used immunoassays. However, a more recent article^17^ showed that new immunoassays overcame this limitation with improved extraction methods, which allows for obtaining accuracy comparable to LC-MS/MS, with a slight overestimation of cortisol levels by immunoassays. Therefore, both methods could be used with comparable specificity; however, because of the difference in the absolute estimated concentrations of cortisol and cortisone, the inter-method comparison of results and establishing reference intervals is more difficult. Moreover, our method requires minimal volume of saliva (200 µl) and is thus easily applicable for collecting repeated cortisol and cortisone measurements in epidemiological studies.

In addition, our method showed high sensitivity to detect a low level of both compounds. Compared to the LC-MS/MS methods published so far, which used the ESI source, we obtained a lower instrumental LOD (in synthetic matrix) for both cortisol and cortisone, which was of 0.014 nmol/L for both compounds and a lower LOQ (0.028 nmol/L for cortisol and 0.138 nmol/L for cortisone)^21,23,30,34–36^. Application of the salivary matrix resulted in a slight increase in the LOQ values (0.28 nmol/L for cortisol and 0.14 nmol/L for conrtisone), which then fall within the range of some of the previously published methods^23,30,31^. However, to our knowledge, the reported LOQ values in these methods were obtained in a synthetic matrix and the effect of the salivary matrix on the LOQ values was not tested. Therefore, we cannot claim that the obtained LOQ in the salivary matrix presents a substantial improvement, but it is certainly of added value because it better approximates the real conditions during sample analysis. Apart from the effect of the salivary matrix on the LOQ values, we observed a minimal effect on the calibration curve itself, which is in line with previous conclusions^21^. In addition, the obtained LOD and LOQ values in the salivary matrix for both compounds were still far below the expected morning levels, which are about 30 and 120 times higher for cortisol and cortisone, respectively^35,36^. We can thus conclude that the calibration in both synthetic and biological matrix (saliva) can be used for CAR assessment with the developed method without a substantial effect on the estimated levels. Still, calibration in biological matrix allows taking into account both the sample preparation and the matrix effect and is therefore recommended.

The developed UPLC-MS/MS method was further validated in sample of healthy volunteers to investigate day-to-day variability of cortisol and cortisone indices. In terms of variability of repeated measurements of cortisol and cortisone, our results indicate high day-to-day variability of both hormones, as reflected by the low ICC values (0.15–0.37 for cortisol and 0.19–0.36 for cortisone). This finding agrees with the literature^10,37–40^. Ross *et al*. reported that more than 50% of the total cortisol index variance in a healthy adult population was due to day-to-day fluctuations and this percentage reached 78% for the CAR values^10^. This situation was also reflected in the low ICC value for CAR, 0.219. Similarly, Almeida *et al*.^37^ obtained an ICC value of 0.22 when investigating intra-individual variability of CAR in a large number of healthy adults. Therefore, our results confirm a high level of short-term, day-to-day fluctuations in CAR levels, which reflect the dynamic activity of the HPA axis.

Moreover, we also observed slight differences in ICC measurements between cortisol and cortisone. For cortisol, the level on awakening was the least stable across different sampling days, with ICC value 0.15, as compared with the 30 min after awakening (ICC = 0.37) or the aggregate measurements (ICC_AUCi_ = 0.29, ICC_AUCg_ = 0.25). This finding contrasts with those of Wang *et al*.^38^, who reported CAR ICC values of 0.31 and 0.17 in two independent studies and a more reliable ICC for the wake-up cortisol levels (0.51 and 0.52). However, in the Wang *et al*. study, CAR was not calculated as AUC but as the difference between cortisol concentrations at 30 and 0 min after awakening, which might explain the observed differences in ICCs. In our study, individual indices of cortisone showed somewhat higher stability (ICC_0min_ = 0.3, ICC_15min_ = 0.28, ICC_30min_ = 0.36) as did the AUCg (ICC = 0.39); the cortisone level increase measured by the AUCi seem to be the least stable (ICC = 0.19). However, to our knowledge, no study assessed day-to-day variability of the morning salivary cortisone, and therefore we cannot make any further comparison of the obtained results.

Furthermore, our results suggest that several confounders need to be taken into account to partially address day-to-day variability of salivary cortisol and cortisone. First, time of awakening had a strong negative impact on all cortisol measurements, except AUCi, which suggests that earlier awakening time corresponds to increased released cortisol level but might not affect the cortisol increase measured by AUCi. This finding agrees with previous research of Edwards *et al*.^41^, showing a negative correlation between time of awakening and the CAR AUCg, although the authors included an additional time point (45 min after awakening), not assessed in the present research, and there was a slight difference in the computation of the AUC formula. In the same study, correlation between time of awakening and AUCi was relatively weak and not robust during 2 days of sampling. Similarly, Pruessner *et al*.^3^ found no significant correlation between time of awakening and AUCi. Moreover, Stalder *et al*. performed a case study over 50 measurement days and found that 38% of the day-to-day variability of cortisol measured immediately after awakening could be explained by the awakening time, but the impact on AUCi was not significant^42^. Therefore, awakening time seems to have more of an impact on the measures of absolute cortisol diurnal secretory activity and is less strongly associated with response to awakening reflected by the AUCi measurements. This hypothesis was also confirmed in previous studies on the effect of individual differences in circadian rhythm on CAR, showing that morning-active individuals who wake early release a higher absolute amount of cortisol in the morning than evening-active individuals^43^.

Second, working day had a negative impact on cortisol AUCg (higher AUCg on working days as compared with weekend), but had no significant effect on AUCi or cortisone aggregate measurements (AUCg and AUCi). The weekday–weekend differences in CAR have been reported previously. Schlotz *et al*.^44^ found a significantly higher CAR on a working day versus a weekend day in 219 healthy people who collected samples on 6 consecutive days. Similarly, Kunz-Ebrecht *et al*.^45^ reported significantly higher AUCg values on working days than weekend days, but the AUCg was calculated as a difference between measurements at 30 and 0 min after awakening, unlike in our study. In both studies, the authors supported these findings with the stress-anticipation hypothesis. According to this hypothesis, our neuroendocrine system adapts to the increased demands present on working days by releasing a higher amount of cortisol^4^. However, in our study, we did not assess anticipated stress and therefore cannot make any further conclusions regarding this link. Nevertheless, the interaction effect of time of awakening and working day on both cortisol and cortisone AUCg values suggests that the effect of the working day can be attributed to additional factors rather than the difference in the awakening hours on weekdays or weekend days only.

In our study, medication use was associated with increased cortisol and cortisone AUCg values, with no effect on AUCi values. The effect of medication use on cortisol levels were thoroughly described in a literature review^46^ suggesting that several groups of medications can affect cortisol levels via different biological mechanisms. This also included non-steroidal anti-inflammatory drugs such as ibuprofen, which were occasionally taken by some of participants in the present study. The simplest and most conservative approach would be to exclude anyone who is taking any medication from participation in the study. However, bearing in mind the small sample size, we used a more strategic approach by assessing medication use as a potential confounder and performing case-by-case assessment in order to identify potential outliers among people who reported taking medication, as suggested in the guidelines for CAR assessment^8^. In addition, unlike some of the previous studies, we found no effect of contraceptive pills on any of the measurements of cortisol and cortisone. However, this finding might be due to lack of power, because only two participants reported taking oral contraception. Finally, we did not observe effects of sex or age on cortisol and cortisone levels; the lack of effect of age might be due to our sample including younger participants with a narrow age range (23–39 years old). Therefore, a larger and more heterogeneous group of participants might have revealed potential effect of age.

There were some limitations to our analyses. First, our study included a small sample of participants (N = 18), which might have led to insufficient power to detect additional significant effects in our mixed models. Nevertheless, this limitation was partially compensated with the repeated sampling of each individual over 1 week, which resulted in a large overall number of measurements. In addition, our sample included a relatively young working population with a narrow age range, so our results might not be representative of a more heterogeneous population. Finally, we did not use any objective verification of awakening time or time of sampling. This, in addition to self-reported data, is an important control for compliance with the sampling protocol because deviations in sampling time can affect the CAR profile and lead to false conclusions^8^. Nevertheless, we gave a thorough explanation to all participants regarding the sampling protocol, the importance of compliance and the possible consequences of incorrect sampling and provided them with take-home written instructions. Moreover, all participants received a diary in which they recorded time of awakening and the exact time when each sample was collected as well as any possible deviation from the protocol. According to the diaries, all participants complied with the sampling protocol, and no deviations were reported. Because concluding on the compliance based on the CAR values and consequent exclusion of participants are not recommended^8^, we did not use this approach in our analysis.

In conclusion, by using a newly developed UPLC-MS/MS method for simultaneous and precise determination of cortisol and cortisone levels in saliva, our study is among the first to describe variability of both levels in a healthy population over 1 week. We observed similar morning patterns of these two hormones, but also high day-to-day variability, which presents a challenge for clinical research. Nevertheless, salivary cortisone level showed somewhat lower intra-individual variability and was less affected by state-like covariates such as time of awakening and working day. Considering these findings, as well as an increasing number of investigations demonstrating the potential of salivary cortisone as an alternative to serum cortisol^20,21^ and as a biomarker of psychological stress^22^, we recommend that future studies further explore the potential of salivary cortisone measurement in psychophysiological stress research. Moreover, bearing in mind the potential time lag between serum cortisol and salivary cortisone level caused by the conversion of cortisol to cortisone by 11β-HSD2^21^, further temporal profiling of morning salivary cortisone level relative to free serum cortisol level is needed to support the use of salivary cortisone as a surrogate marker.

## Supplementary information

Supplementary information

## Data Availability

All data generated or analysed during this study are included in this published article.
