# Supplementary material for: Study of temporal variability of salivary cortisol and cortisone by LC-MS/MS using a new atmospheric pressure ionization source
Source: Sci Rep. 2019 Dec 17;9:19313. doi: 10.1038/s41598-019-55571-3 (PMC6917784; doi:10.1038/s41598-019-55571-3)
Supplement: Supplementary file 1 — Supplementary information [file 41598_2019_55571_MOESM1_ESM.pdf]

Study of temporal variability of salivary cortisol and cortisone by LC-MS/MS using a new atmospheric pressure ionization source

Jelena Bakusic[1*], Siemon De Nys[2], Matteo Creta[1], Lode Godderis[1,3], Radu Corneliu Duca[1,4]

[1] Environment and Health, Department of Public Health and Primary Care, KU Leuven, Kapucijnenvoer 35, blok d – box 7001, 3000 Leuven, Belgium. jelena.bakusic@kuleuven.be, matteo.creta@kuleuven.be, lode.godderis@kuleuven.be, radu.duca@kuleuven.be

[2] Department of Oral Health Sciences, BIOMAT & University Hospitals Leuven (UZ Leuven), Dentistry, Kapucijnenvoer 7, blok a – box 7001, 3000 Leuven, Belgium. siemon.denys@kuleuven.be

[3] IDEWE, External Service for Prevention and Protection at Work, Heverlee, Belgium

[4] Unit Environmental Hygiene and Human Biological Monitoring, Department of Health Protection, National Health Laboratory, 1, Rue Louis Rech, L-3555 Dudelange, Luxembourg

Corresponding authors:

Jelena Bakusic, MD

Centre for Environment and Health,

Department of Public Health and Primary Care,

KU Leuven (University of Leuven),

Kapucijnenvoer 35, 3000 Leuven,

Belgium

E-mail: jelena.bakusic@kuleuven.be

Radu Corneliu Duca, PhD

Unit Environmental Hygiene and Human Biological Monitoring

Department of Health Protection

National Health Laboratory

1, Rue Louis Rech,

L-3555 Dudelange,

Luxembourg

E-mail: radu.duca@lns.etat.lu

Supplementary information

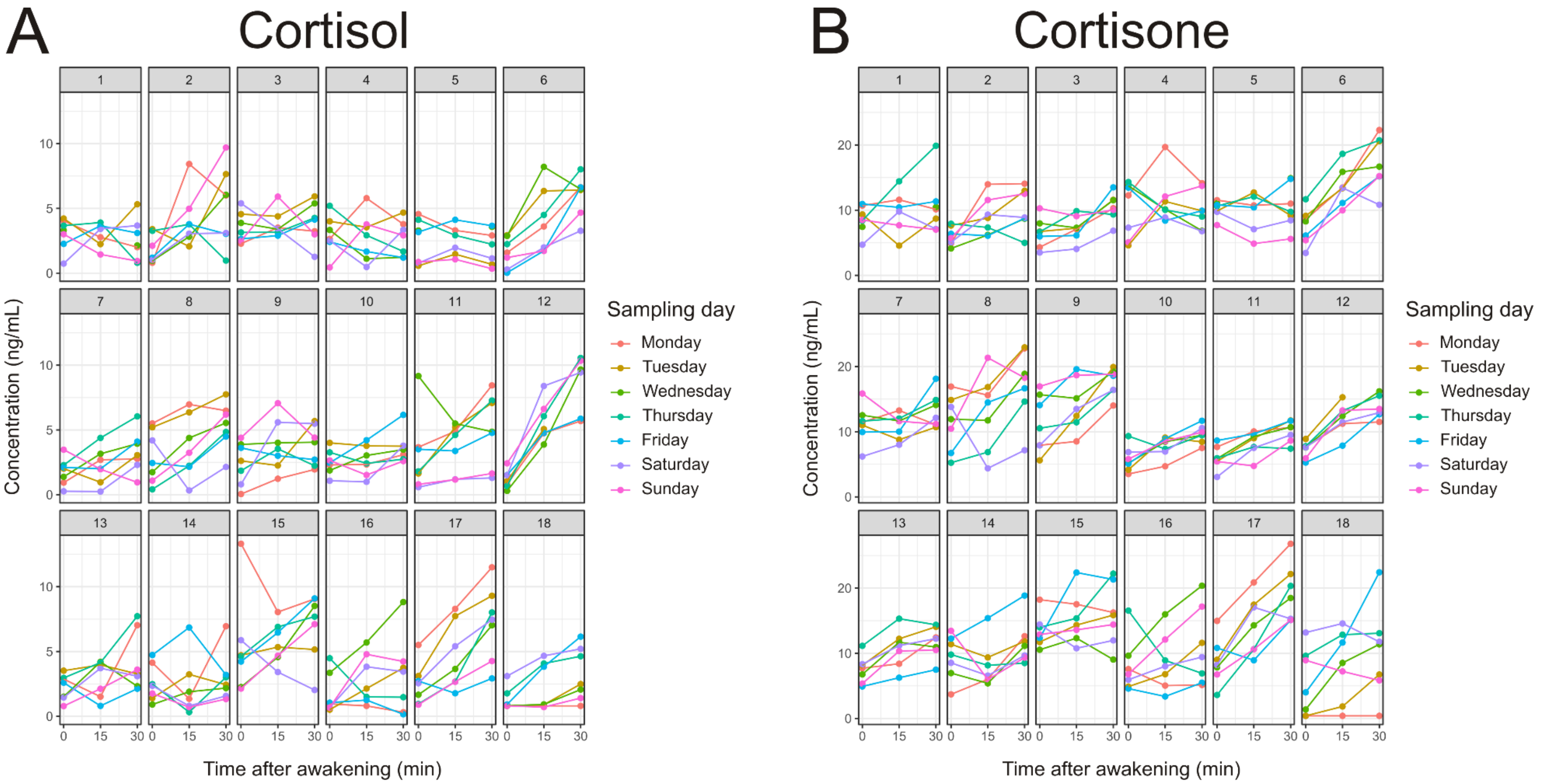

Supplementary Figure 1. Overview of cortisol (A) and cortisone (B) concentrations of each participant obtained on each day over one sampling week at 0, 15 and 30 min after awakening
